# The predictive roles of parental stress and intolerance of uncertainty on psychological well-being of parents with a newborn in neonatal intensive care unit: a hierarchical linear regression analysis

**DOI:** 10.1186/s12887-023-04420-4

**Published:** 2023-12-01

**Authors:** Masoume Rambod, Nilofar Pasyar, Zeinab Mazarei, Mitra Soltanian

**Affiliations:** 1 Community Based Psychiatric Care Research Center, Nursing and Midwifery School, Shiraz University of Medical Sciences, Shiraz, Iran; 2 Student Research Committee of Shiraz University of Medical Sciences, Shiraz, Iran

**Keywords:** Parents, Uncertainty, Psychological well-being, Intensive care units, Neonatal

## Abstract

**Background:**

Hospitalization of newborns in neonatal intensive care units (NICU) exposes parents to considerable stress. This study aimed to determine the predictive role of parental stress and intolerance of uncertainty on the psychological well-being of parents with a newborn in NICU using hierarchical linear regression analysis.

**Methods:**

This cross-sectional study included 130 parents of newborns hospitalized in the NICU. Data were collected using Parental Stress Scale, Intolerance of Uncertainty Scale, and Psychological Wellbeing Scale. The data were analyzed in SPSS v.26 using hierarchical linear regression analysis.

**Results:**

The mean scores of the parents’ psychological well-being, parental stress, and intolerance of uncertainty were 79.08 (SD = 11.70), 63.06 (SD = 26.71), and 75.38 (SD = 19.40), respectively. The result of the hierarchical linear regression analysis revealed that, in step 1, academic education had a significant proportion of the variance of parents’ psychological well-being (β = 0.26, P = 0.005). In step 2, it was shown that academic education (β=-0.25, P = 0.006) and parental stress (β=-0.25, P = 0.006) had a significant proportion of the variance of parents’ psychological well-being. According to step 2, education levels, parental stress, and intolerance of uncertainty explained 22% of the changes in parents’ psychological well-being.

**Conclusion:**

There was a significant association between the parents’ psychological well-being and education levels, intolerance of uncertainty, and parental stress. Academic education and parental stress were the predictors of parents’ psychological well-being. Based on these findings, early detection of parents’ education and stress are important for their psychological well-being.

## Introduction

In recent years, the number of newborn babies admitted in hospital has increased [1]. When a newborn is admitted in hospital, especially in neonatal intensive care unit (NICU), his/her family experiences a great deal of stress. If these stressors are not decreased, both the family and the baby’s health will be affected [2].

The parents of the newborn hospitalized in NICU are exposed to some stressors such as complicated physical environment and medical equipment and its noise. Moreover, the newborn admitted in NICU had common health conditions such as apnea, broncho-pulmonary dysplasia, respiratory distress syndrome, etc. Some of them need endotracheal tube, gastric tube feeding, oxygen therapy, etc. These tubes and devices can trigger stress in their parents because of their appearance, fragility, and behavior [3]. The changes in parental role, financial burden, appearance and behavior of the baby, and light and sounds in the environment have been identified as the most stressful factors that cause negative feelings in parents [4, 5]. A study indicated that mothers with premature and low birth weight newborns admitted in NICU had higher scores of acute stress disorder compared to those with full-term newborns [6]. Another study reported that sudden noise in NICU led to anxiety in parents [7]. In fact, NICU parental stress is associated to mothers and fathers’ anxiety and depression [8].

The routine leisure activities, interpersonal interactions of the family, and different aspects of the parents’ well-being are significantly affected when a baby is born. All these changes, however, are more pronounced and impose an additional psychological burden on family members when a baby is delivered with medical problems, such as abnormalities [9]. Disappointment, guilt feeling, depression, anger, fear, anxiety, sense of failure, and loss of self-confidence are some of the most frequent psychological responses of parents to the hospitalization of their newborns in the NICU [10].

Uncertainty is one of the sensations shared by parents of babies admitted to the NICU. Parental uncertainty in illness of a newborn is defined as “a paradoxical, cognitive and emotional experience in which there is an inability to create meaning and may cause disruption in parental role development” [11]. The intolerance of uncertainty is a common occurrence in parents of newborns in NICU. Several variables, including parental literacy levels, socioeconomic status, emotional reactions, cultural traits, and the relationship between parents and healthcare professionals can influence the development of parental uncertainty [12]. According to Dar et al., people who cannot tolerate uncertainty find ambiguity stressful, frustrating, and anxiety [13]. Uncertainty can also be a significant source of distress, affecting parents’ adjustment and well-being [14].

It was reported that admission of the newborns in NICU for two weeks affected psychological well-being and led to experience of depression, anxiety, and stress in the parents [15]. It was demonstrated that family psychological health and anxiety are predicted by parents’ psychological well-being. As the first persons a newborn forms a bond with, parents—especially mothers—play a crucial role in the development of the child’s psychological and emotional traits and are viewed as the source of health or disease. This highlights the need to offer the family nursing care, medical services, and social support, which should continue during hospitalization and even after the newborn has been discharged [16].

The materials mentioned above demonstrate that some factors may influence parents’ well-being while their baby is receiving medical treatment in the NICU. Few studies have been conducted on parents’ uncertainty [12, 13], psychological well-being [17, 18], mental health [19], and stress [20–22] in parents with newborns in NICU and factors associated to it. However, a limited number of studies have examined the association between psychological well-being and uncertainty intolerance and parental stress in parents with a newborn in NICU. Moreover, the predictors of psychological well-being in these parents are unknown. In addition, there is limited evidence regarding the impact of adding variables such as intolerance of uncertainty and parental stress in the regression model to determine the predictors of parents’ psychological well-being. Therefore, to improve the evidence-based practice, in the current study we aimed to evaluate the predictive roles of intolerance of uncertainty and parental stress on the well-being of parents with a newborn hospitalized in the NICU. The specific aims were as follows:

1. The association of parental psychological well-being with demographic and clinical characteristics of newborns and parents.
2. The association of parents’ psychological well-being and parental stress and intolerance of uncertainty with a newborn in NICU.
3. Determination of parental stress and intolerance of uncertainty as the predictor of psychological well-being of parents with a newborn in NICU, using hierarchical linear regression analysis.

## Method

### Design

This cross-sectional study was conducted on 130 parents of newborns hospitalized in NICU.

### Setting

The setting of this study was Hazrat Valiasr Hospital in Kazeroon, Iran.

### Participants

This study was conducted on parents who had a newborn in NICU. At least three days of admission in the newborn was passed in NICU. The parents should be able to read and write, as well as speak and comprehend the Persian language. The parents who had a history of psychological diseases based on their reports and their newborns had died in NICU were excluded.

### Sample size

Due to the lack of an article on all the parents with newborns hospitalized in the NICU and study title, the sample size was calculated to be 130 participants using the pilot study. Following data collection and data analysis, a power analysis was used to determine the adequacy of the sample size. Based on 130 participants, the correlation coefficient between parental psychological well-being and uncertainty intolerance = -0.29, and α = 0.05, the power (1- β) was determine as 92%. Therefore, this power showed that the sample size was adequate.

### Measurements

The parents and their newborns’ demographic and clinical characteristics form, Parental Stress Scale, Intolerance of Uncertainty Scale, and Psychological Wellbeing Scale were used to collect the data. The parents and their newborns’ demographic and clinical characteristics form included questions regarding the parents’ age, gender, marital status, employment status, education level, number of their children, history of newborns hospitalized in NICU, type of mother delivery, pregnancy type (wanted, unwanted), comorbidities, and history of drug administration. The clinical information form for the newborn also contained questions on the newborns’ gender, diagnosis, and duration of his/her stay in the hospital.

Parental Stress Scale was developed by Miles in 1987. The scale with 34 items and three subscales including sights and sounds (6 items), neonate’s behavior and appearance (17 items), and parenteral role alternation (11 items) was used. The range of each item using a Likert scale was from 1 (not stressful) to 5 (extremely stressful). There is also a “not applicable” option that is scored 0. More stress equals a higher score [23]. The concurrent validity of the scale with State Anxiety Scale was in the moderate level (0.20–0.44). The Cronbach’s alpha coefficient was used in the preliminary analysis by Miles et al. to determine the reliability of the scale for each subscale and the total scale. The reliability of the total scale was determined 0.94. The Cronbach’s alpha for their subscales was from 0.73 to 0.92 [23]. With a Cronbach’s alpha of 0.86, the reliability was verified [24]. In this study, Cronbach’s alpha for the total scale, sights and sounds, neonate’s behavior and appearance, and parenteral role alternation subscales were 0.94, 0.78, 0.93, and 0.89, respectively.

Intolerance of Uncertainty Scale developed by Freeston (1994) was used. It has 27-items scored using a 5-point Likert scale ranging from 1 (characteristic of me not at all) to 5 (entirely characteristic of me). The scores are between 27 and 135 [25]. It assesses four scales of “unexpected events and should be avoided”, “uncertainty is stressful and upsetting”, “uncertainty leads to the inability to act”, and “being uncertain about the future is unfair” [26]. A score of 54 is the cut-off point of the scale, and scores less than 54 demonstrate intolerance of uncertainty at a low level; also, scores higher than that demonstrate intolerance of uncertainty at a high level. Cronbach’s alpha of this scale was approved by α = 0.91, and the test- retest reliability with a 4-week interval was reported as r = 0.78 initial French version. The validity of this scale was acceptable [25]. It was revalidated in the research conducted by Buher and Dugus in 2002, and the Cronbach’s alpha and test-retest reliability after 5 weeks for this scale were reported as 0.94 and 0.74, respectively [26]. In another study, the convergent validity of the scale was approved by “Penn State Worry Questionnaire”, “Generalized Anxiety Disorder 7-Item Scale”, and “State-Trait Inventory for Cognitive and Somatic Anxiety” [27]. According to Moradi and Jafari’s research, the reliability of the intolerance of uncertainty scale was 0.78 [28]. In this study, Cronbach’s alpha for the total Intolerance of Uncertainty Scale, “unexpected events and should be avoided”, “uncertainty is stressful and upsetting”, “uncertainty leads to the inability to act”, and “being uncertain about the future is unfair” subscales were 0.92, 0.76, 0.79, 0.85, 0.75.

Psychological Wellbeing Scale designed by Ryff in 1989 was used. It was an 18-item psychological well-being scale. This scale consists of six subscales including autonomy, environment mastery, personal growth, positive relations with others, purpose in life, and self-acceptance. It is scored using a seven-point Likert scale ranging from strongly disagree (n = 1) to strongly agree (n = 7). The higher the score, the higher the psychological well-being [29]. The short form of Ryff’s psychological well-being scale showed a correlation with the original scale ranging from 0.7 to 0.89, and its factor analysis validity and convergent validity have been validated with other mental health scales. Khanjani et al. found that the internal consistency of this scale was 0.71 using Cronbach’s alpha, which agrees with that of Ryff [30]. In this study, Cronbach’s alpha for the total scale was approved 0.78.

### Data collection

After permission of Shiraz University of Medical Sciences (SUMS), the manager of Hazrat Valiasr Hospital, and the NICU head nurse, the data collection was conducted. It was performed by a researcher assistant with a work experience as a nurse for six years in NICU. The data were collected using convenience method. To this end, the parents who had inclusion criteria and were accessible and available to the researcher assistant in the NICU were selected. Then, they were enrolled in the study and filled out the questionnaires.

### Ethical considerations

The Ethics Committee of SUMS approved this study (IR.SUMS.NUMIMG.REC.1400.010). The permission of this study was approved by SUMS, Hazrat Valiasr Hospital, and NICU. Written informed consent was signed by the parents who had a newborn in NICU. All participants confirmed the necessary cooperation to perform the study, which included information on the research aims, participants’ activities, the time required to complete the questionnaire, and the voluntary nature of participation in the research. The participants were assured that their information would remain confidential and that the concept of anonymity would be followed.

### Statistical analysis

SPSS for Windows version 22 was used for statistical analysis. To evaluate psychological well-being, parental stress, and uncertainty intolerance, we used descriptive statistics (mean and standard deviation). Independent t-test and ANOVA were used. Pearson’s correlation coefficient was used to assess the correlation between research variables. To conduct multiple linear regression, we assessed the assumptions (autocorrelation, normality, etc.). To assess autocorrelation, we used Durbin-Watson test. Based on Durbin-Watson test that ranged 0–4, a value of 2 or nearly 2 showed no autocorrelation. Multicollinearity test using variance inflation factor (VIF) was used to determine whether there was a strong association among the independent variables. In addition, hierarchical linear regression analysis was applied to identify the variables that predicted the parents’ psychological well-being. Based on the researchers’ experience, “cause of newborn hospitalization” might be a covariate and affect the psychological well-being, uncertainty intolerance, and parental stress. However, ANOVA analysis showed these associations were not significant (p > 0.05) and “cause of newborn hospitalization” was not a covariate in this study. A p-value < 0.05 was considered as the statistical significance.

## Results

This study included the parents of 130 newborns hospitalized in the NICU. The majority of subjects were female (67.7%). Half of the fathers and 43.2% of the mothers were 21–30 years old. In addition, 39.2% of the parents had high school and diploma education, and 53.8% were housewives. Moreover, 99.2% of the parents were living together. No parent had a history of premature birth. The majority of the parents (84.6%) had no history of comorbidities conditions, and the majority (86.9%) had no history of drug administration (Table 1).

**Table 1:** Demographic and clinical characteristics of the parents and their association with parental psychological well-being

Variables	n (%)	Parental psychological well-being Mean ± SD
Parents gender		
Female Male	88 (67.7) 42 (32.3)	78.93 ± 10.92 79.41 ± 13.37
Test, P-value		t ^a^=-0.21, P = 0.82
Father’s age groups		
≤ 20 21–30 31–40 ≥41	2 (4.8) 21 (50.0) 16 (38.1) 3 (7.1)	72.0 ± 7.07 78.00 ± 13.06 83.53 ± 14.69 73.66 ± 9.07
Test, P-value		F ^b^=0.93, P = 0.43
Mother’s age groups		
≤ 20 21–30 31–40 ≥41	3 (3.4) 38 (43.2) 42 (47.7) 5 (5.7)	71.00 ± 12.76 78.89 ± 11.86 79.57 ± 9.51 79.57 ± 9.51
Test, P-value		F ^b^ =0.56, P = 0.63
Parents’ marital status		
Lived together A divorced single parent	129 (99.2) 1 (0.8)	79.00 ± 11.72 89.00
Test, P-value		t ^a^ =-0.84, P = 0.39
Parents’ education level		
Primary school Middle school High School and Diploma Academic	21 (16.2) 28 (21.5) 51 (39.2) 30 (23.1)	75.57 ± 6.59 75.85 ± 10.60 78.43 ± 12.38 85.89 ± 12.02
Test, P-value		**F** ^b^ **=5.11, 0.002**
Parent’s jobs		
Housewife Employed Unemployed Retired	70 (53.8) 51 (39.2) 7 (5.4) 2 (1.5)	77.28 ± 11.92 77.67 ± 10.62 69.50 ± 0.70 81.70 ± 12.96
Test, P-value		F ^b^ =1.70, P = 0.17
Parents’ comorbidities condition		
Yes No	20 (15.4) 110 (84.6)	82.90 ± 10.16 78.38 ± 11.87
Test, P-value		t ^a^ =1.59, P = 0.11
Parents’ drug administration		
Yes No	17 (13.1) 113 (86.9)	82.11 ± 9.24 78.62 ± 12.00
Test, P-value		t ^a^ =1.14, P = 0.25
Previous history of premature birth		
Yes No	0 (0.0) 130 (100.0)	Cannot be computed
Test, P-value		

^a^ independent t- test, ^b^ ANOVA

As Table 2 shows, prematurity (40%) and respiratory distress (34.6%) were the most prevalent reasons for the admission of newborns in NICU. Moreover, 53.8% of the newborns were the first baby of their parents. In addition, 63.8% of the newborns were born by cesarean section.

**Table 2:** Demographic and clinical characteristics of the newborns and their association with parental psychological well-being

Variables	n (%)	Parental psychological well-being Mean ± SD
Cause of newborn hospitalization		
Premature Respiratory distress Sepsis Low Apgar score Cyanosis Seizure Jaundice Intolerance of feeding Apnea Surgery	52 (40.0) 45 (34.6) 11 (8.5) 6 (4.6) 5 (3.8) 4 (3.1) 2 (1.5) 2 (1.5) 2 (1.5) 1 (0.8)	79.96 ± 12.28 81.08 ± 10.84 74.72 ± 11.10 72.40 ± 14.97 68.60 ± 9.63 76.75 ± 10.04 91.00 ± 2.82 69.00 ± 2.82 83.50 ± 9.19 74.00
Test, P-value		F ^a^=1.49, 0.15
Birth order		
First Second Third Fourth Fifth	70 (53.8) 40 (30.8) 16 (12.3) 3 (3.12.3) 1(0. 8)	78.08 ± 11.46 79.05 ± 11.53 82.43 ± 12.51 87.66 ± 16.16 70.00
Test, P-value		F ^a^=1.00, P = 0.40
Type of birth delivery		
Cesarean Vaginal	83 (63.8) 47 (36.2)	79.97 ± 11.98 77.53 ± 11.16
Test, P-value		t ^b^=1.14, P = 0.25
Type of pregnancy		
Wants Unwanted	114 (87.7) 16 (12.3)	79.37 ± 12.04 77.06 ± 9.00
Test, P-value		t ^b^ =0.73, 0.46

^a ANOVA, b Independent t−test^

### The mean scores of parental’ stress, intolerance of uncertainty, and psychological well-being

The parents’ mean score of stress was 63.06 ± 26.71, which ranged from 9 to 117. Table 3 shows the mean scores of parental stress subscales. The mean score of parental intolerance of uncertainty was 75.38 ± 19.40, which ranged from 31 to 125. Table 3 shows the mean scores of parental intolerance of uncertainty subscales. Among the parents, 23 (17.7%) and 107 (82.3%) reported lower and higher levels of intolerance of uncertainty, respectively.

**Table 3:** The mean scores of the parents’ intolerance of uncertainty, stress, and psychological well-being

Variables	Min-Max	Mean ± SD
**Intolerance of Uncertainty**	31–125	75.38 ± 19.40
Dimensions of intolerance of uncertainty		
Uncertainty leads to the inability to act	11–48	25.47 ± 7.71
Uncertainty is stressful and upsetting	11–45	26.96 ± 6.63
Unexpected events and should be avoided	5–23	12.76 ± 3.97
Being uncertain about the future is unfair	4–20	10.17 ± 3.39
**Parental stress**	9-117	63.06 ± 26.71
Dimensions of parental stress		
Sights and sounds	0–28	12.27 ± 5.25
Neonate’s behavior and appearance	0–61	30.89 ± 16.43
Parenteral role alternation	0–44	19.88 ± 10.48
**Psychological well-being**	57–110	79.08 ± 11.70
Dimensions of psychological well-being		
Autonomy	6–19	12.26 ± 2.84
Environment mastery	6–16	12.15 ± 2.09
Personal Growth	7–20	13.69 ± 3.36
Positive relations with others	7–21	14.28 ± 2.82
Purpose in life	5–20	12.54 ± 3.22
Self-Acceptance	6–21	14.26 ± 3.18

The parents’ mean score of psychological well-being was 79.08 ± 11.70 with a range of 57–110. Table 3 shows the mean scores of parental psychological well-being subscales. Among several aspects of psychological well-being, the lowest mean score belonged to “environment mastery” which was 12.15 ± 2.09, and the highest mean score was 14.28 ± 2.82 for the dimension of “positive relations with others” (Table 3).

### Aim 1. The association of parental psychological well-being and demographic and clinical characteristics of newborns and parents

As Tables 1 and 2 show, no significant difference was observed between parental psychological well-being and all parents and newborns’ demographic and clinical characteristics expect for parents’ education level. The result of ANOVA showed that parents with academic education had the highest psychological well-being (F = 5.11, 0.002). Tukey’s HSD test indicated a significant difference between academic education and primary school and parents’ psychological well-being (Mean difference = 10.32, SD. Error = 3.20, p = 0.009). A significant difference was found between academic education and secondary school and parents’ psychological well-being (Mean difference = 10.03, SD. Error = 2.96, p = 0.005). In addition, a significant difference was observed between academic education and high school and parents’ psychological well-being (Mean difference = 7.46, SD. Error = 2.60, p = 0.02).

### Aim 2. The association of parents’ psychological well-being and parental stress and intolerance of uncertainty

The Pearson correlation coefficient test showed a negative and significant, but a weak, association between the participants’ psychological well-being and their parental stress (r=-0.31, p < 0.001) and all its subscales. Parental psychological well-being decreased as parental stress increased. In addition, the Pearson correlation coefficient test revealed a negative and significant, but weak, association between the parents’ psychological well-being and total intolerance of uncertainty (r=-0.27, p = 0.002) and all of its subscales (P < 0.05). As a result, when parents’ intolerance of uncertainty increased, their psychological well-being decreased (Table 4).

**Table 4:** The association between parental psychological well-being and intolerance of uncertainty and stress

		Parental psychological well-being	
**Intolerance of uncertainty**		r ^a^=-0.27, P = 0.002	
Dimensions of intolerance of uncertainty			
Uncertainty leads to the inability to act		r ^a^ =-0.28, P = 0.001	
Uncertainty is stressful and upsetting		r ^a^ =-0.23, P = 0.006	
Unexpected events and should be avoided		r ^a^ =-0.18, P = 0.03	
Being uncertain about the future is unfair		r ^a^ =-0.21, P = 0.01	
**Parental stress**		r ^a^ =-0.31, P < 0.001	
Dimensions of parental stress			
Sights and sounds		r ^a^ =-0.24, P = 0.004	
Neonate’s behavior and appearance		r ^a^ =-0.23, P = 0.008	
Parenteral role alternation		r ^a^ =-0.31, P < 0.001	

r ^a^: Pearson correlation coefficient (r)

### Aim 3. Hierarchical linear regression analysis used to determine the predictor of parents’ psychological well-being

The normality of psychological well-being, parental stress, and uncertainty intolerance was approved and the p-value of Kolmogorov-Smirnov test were > 0.05. In this study, Durbin-Watson statistic was 1.98, with a range of 1.5–2.5, which was acceptable. As Table 5 shows, Collinearity statistics (VIF) of the variables were smaller than 10; there was no multicollinearity in the independent variables of this study.

**Table 5:** Hierarchical linear regression analysis of education level, intolerance of uncertainty, and parental stress in prediction of parental psychological well-being

	Step 1				Step 2			
	β	t	p	Collinearity statistics (VIF)	β	t	p	Collinearity statistics (VIF)
Constant		49.98						
Primary school Secondary school Academic education	-0.1 -0.09 0.26	-1.09 -0.97 2.86	0.27 0.33 0.005	1.18 1.21 1.21				
Constant						21.95		
Primary school Secondary school Academic education Parental stress intolerance of uncertainty					-0.75 -0.12 0.25 -0.25 -0.13	-0.86 -1.38 2.82 -2.78 -1.40	0.39 0.16 0.006 0.006 0.16	1.20 1.23 1.23 1.32 1.39
R	0.33				0.47			
R^2^	0.11				0.22			
Adjust R^2^	0.09				0.19			
R^2^ change	0.11				0.11†			
F	5.20				7.10			

†R^2^ Step 2- R^2^ Step 1

As Table 5 shows, hierarchical linear regression analysis was used to determine the predictors of parents’ psychological well-being. As only parents’ education level was associated to parents’ psychological well-being in the univariate analyses, it was entered in step 1 of multiple regression analysis. In step 1, academic education had a significant proportion of the variance of parents’ psychological well-being (β = 0.26, P = 0.005). Parents’ psychological well-being was at the highest level in parents with academic education compared to other parents (R = 0.33, R^2^ = 0.11, Adjust R^2^ = 0.09). In step 2, education level, parental intolerance of uncertainty and stress were entered. It showed that academic education (β=-0.25, P = 0.006) and parental stress (β=-0.25, P = 0.006) had a significant proportion of the variance of parents’ psychological well-being. Parents with academic education lower stress reported better psychological well-being (R = 47, R^2^ = 0.22, Adjust R^2^ = 0.19). However, intolerance of uncertainty did not predict parental psychological well-being (β=-0.08, p = 0.16). According to step 2, education level, parental stress and intolerance of uncertainty explained 22% of the changes in parents’ psychological well-being.

## Discussion

To the best of our knowledge, this is the first study conducted on parents with a newborn admitted in NICU, which aimed to evaluate the roles of parental stress and intolerance of uncertainty in prediction of parental psychological well-being. The findings showed parental stress and academic education were predictors of parental psychological well-being.

The findings of the current study revealed that parents’ mean score of stress was 63.06. In the same line with our study, Heidari et al. found that 73.8% of parents whose newborns were hospitalized in NICU experienced severe stress [31]. Moreover, Sisodia et al. indicated that parents experienced moderate levels of stress [32]. Another study showed that the rate of stress was high in premature newborn’ parents [8]. Every parent wishes to have a healthy child. During pregnancy, parents expect and wish to have a healthy and mature baby. When a premature and unwell newborn is born, parents suffer acute tension and anxiety over the insecurity of their newborn’s condition because of the unexpected event and shift in their parental position [33]. The disturbance of the parental role, the ward’s physical environment, and the baby’s pain are the main sources of emotional stress for mothers of newborns admitted to NICU [34]. It was reported that stress of “the sights and sounds in NICU”, “the looks of my baby”, and “my inability to be a parent” were the maternal stress experienced by mothers when their newborns were hospitalized in NICU [35].

The results of this study showed that the mean score of parental intolerance of uncertainty was 75.38. Moreover, among the parents, 82.3% reported a high level of intolerance of uncertainty. According to Montalvo et al., in a study entitled ‘uncertainty associated to parents of preterm newborns hospitalized in neonatal intensive care’, both fathers (52.6%) and mothers (49.3%) reported a high level of uncertainty [36]. Uncertainty in parents of newborns in the NICU had a direct and indirect effect on post-traumatic stress disorder [37]. A psychological trait called intolerance of uncertainty is characterized by intense fear and a lowered capacity to deal with the unknown [38]. High levels of intolerance of uncertainty are characterized by negative interpretations of uncertain situations, the belief that one cannot handle uncertainty and emotional discomfort [31].

In the current study, the score of psychological well-being was 79.08. Among the several aspects of psychological well-being, the lowest and highest mean score was related to “environment mastery”, and “positive relations with others”, respectively. In the studies of Amiri et al. [39] and Tandberg et al. [18], parents reported an average amount of psychological well-being. One of the most fundamental concepts in positive psychology is well-being. A high amount of well-being is a state in which a person is in a good and optimal state of functioning. People who have a high sense of well-being are less likely to suffer from sadness and anxiety [40]. From a psychological point of view, parents of newborns admitted to the NICU are a susceptible group because they suffer trauma (due to critical delivery and separation from their baby) and stress (related to medical issues and accompanying interventions) [18, 41].

In this study, the parents’ education level was associated to their psychological well-being and parents who had academic education reported better psychological well-being. A study indicated that educational level was one of the factors associated to parental satisfaction [42, 43].

The current study found a significant and negative association between parental psychological well-being and intolerance of uncertainty and parental stress using the univariate analyses. Consistent with this study, it was reported that parental psychological symptoms indicated significant effects on intolerance of uncertainty [44]. Parental intolerance of uncertainty during a child illness was attributed to parents’ emotional (fear, hopelessness, and feeling overwhelmed) and cognitive (psychological adjustment and health behaviors) experiences [11].

The study findings revealed that parental stress was a predictor of parents’ psychological well-being. In the first step of hierarchical linear regression analysis, education levels had a significant proportion of the variance of parents’ psychological well-being (11%). In the second step, education levels, parental stress, and intolerance of uncertainty explained 22% of the changes in parents’ psychological well-being. In fact, parental stress and intolerance of uncertainty increased 11% of variance of parents’ psychological well-being. Consistent with this study, the research on young adults showed that stress was one of the predictors of psychological well-being [45]. According to Dar et al., individuals with a high tolerance for uncertainty may find it difficult to deal with stressful daily situations that entail varying levels of uncertainty; as a result, they are more likely to suffer high levels of stress [13]. Rolle et al. demonstrated that mental health was a mediator between parental stress and dynamic adjustment [46]. According to the findings of the current study, parents’ psychological well-being declined as their ability to tolerate uncertainty increased. The findings of a study showed how intolerance for uncertainty had an impact on psychological well-being [47]. As a result, it is possible to regard intolerance for uncertainty in the present as a significant risk factor for affecting psychological well-being [47]. Parenting stress in the NICU is crucial for both the mental health of the parents as well as the potential effects on their relationship with their newborns and the child’s development down the road even though the NICU provides the best care possible for the newborns. To ensure the family’s well-being and avert future issues, we need to pay close attention to the emotional experiences of parents in the NICU from the first moments after delivery and to offer the necessary support, especially to the more vulnerable parents. For optimizing the child’s health and the psychological well-being of parents in this incomparable era, strategies to reduce it are required to identify and formulate coherent and effective solutions for enhancing the psychological well-being of parents. By providing opportunities for parents to be able to care for their premature and sick babies in NICU, parents’ stress can be reduced, and they would be empowered [41, 48, 49].

### Clinical implications

This study provided new clinical information regarding parental academic education and stress which predicted the psychological well-being of the parents whose newborns were admitted in NICU. Therefore, these findings help the NICU professionals and parents to improve the parents’ psychological well-being. Moreover, the clinical implication of this study is that it may be useful for performing educational interventions regarding healthy and sick newborns, sights and sounds, neonate’s behavior and appearance, and parenteral role alternation when the newborn is hospitalized in NICU. This intervention may improve the parents’ psychological well-being.

### Strengths, limitations and future studies

Assessment of three psychological concepts including parental stress, intolerance of uncertainty, and psychological well-being of parents with a newborn in NICU in one research was one of the strengths of this study. The concepts were collected at one time period, and there was no need to follow up and there were no missing data. Therefore, it was another strength of this study. Using hierarchical linear regression analysis enabled us to evaluate the impact of adding variables such as education levels, intolerance of uncertainty, and parental stress in the regression model. Therefore, it was possible to know the contribution of each variable in the model.

One hundred and thirty parents whose newborns were admitted in NICU participated in this study. Although the power of this study was 0.92, a larger sample size in different NICUs around the world is recommended to improve the generalizability of this study. As this is a cross-sectional study, it was not possible to find the causal inference, which is a fundamental limitation of this study. Therefore, future studies to determine the causality between the variables are suggested. In addition, a longitudinal study to assess the parents’ and children’ outcomes of intolerance of uncertainty and stress, and psychological well-being of parents with a newborn in NICU is recommended. Future qualitative studies to assess intolerance of uncertainty, stress, and psychological well-being of parents with a newborn in NICU are suggested. This study indicated that 22% of the variance of parents’ psychological well-being was determined by the variables assessed in this study. On the other hand, other variables determined 78% of the parents’ psychological well-being variance. Therefore, further studies are recommended to be conducted to clarify the relationship of these variables.

## Conclusion

The findings of this study indicated that 82.3% of the parents with a newborn in NICU had a high level of intolerance of uncertainty. The mean scores of parental stress and psychological well-being were 63.06 and 79.08, respectively. The study findings demonstrated the association of the parent’s psychological well-being, education level, intolerance for uncertainty and parental stress. Furthermore, parental stress and academic education were the predictors of parents’ psychological well-being. Therefore, early detection of parents’ education and stress is important for their psychological well-being. Given that 22% of the variance of parents’ psychological well-being was determined by the variables assessed in this study, another study to recognize other predictors of parents’ psychological well-being is suggested.

## Data Availability

The data transparency was approved by NP, ZM and MR. Data are available by MR, NP, and ZM through SPSS file. Data would be available on request based on the privacy and ethical limitations to contact to Nilofar Pasyar (pasyarn@yahoo.com).
